# Objective Acoustic Quantification of Phonatory Dysfunction in Huntington's Disease

**DOI:** 10.1371/journal.pone.0065881

**Published:** 2013-06-10

**Authors:** Jan Rusz, Jiří Klempíř, Eva Baborová, Tereza Tykalová, Veronika Majerová, Roman Čmejla, Evžen Růžička, Jan Roth

**Affiliations:** 1 Department of Circuit Theory, Czech Technical University in Prague, Faculty of Electrical Engineering, Prague, Czech Republic; 2 Department of Neurology and Centre of Clinical Neuroscience, Charles University in Prague, First Faculty of Medicine, Prague, Czech Republic; University of Iowa Carver College of Medicine, United States of America

## Abstract

**Purpose:**

Although speech motor changes are reported as a common sign of Huntington’s disease (HD), the most prominent signs of voice dysfunction remain unknown. The aim of the current study was to explore specific changes in phonatory function in subjects with HD.

**Method:**

34 subjects with HD and 34 age- and sex-matched healthy controls were examined. Participants performed sustained vowel phonation for subsequent analyses of airflow insufficiency, aperiodicity, irregular vibrations of vocal folds, signal perturbations, increased noise, and articulation deficiency. In total, 272 phonations were collected and 12 voice parameters were extracted. Subsequently, a predictive model was built to find the most salient patterns of voice disorders in HD. The results were also correlated with disease severity according to the Unified HD Rating Scale (UHDRS) motor score.

**Results:**

Subjects with HD showed deterioration in all investigated phonatory functions. Irregular pitch fluctuations, sudden phonation interruption, increased noise, and misplacement of articulators were found to be most significant patterns of phonatory dysfunction in HD (*p*<0.001). The combination of these four dysphonia aspects contributed to the best classification performance of 94.1% (sensitivity: 95.1%; specificity: 93.2%) in the separation of HD patients from healthy participants. Our results further indicated stronger associations between sudden phonation interruption and voluntary components of the UHDRS (*r* = −0.48, *p*<0.01) and between misplacement of articulators and involuntary components of the UHDRS (*r* = 0.52, *p*<0.01).

**Conclusions:**

Our configuration of phonatory features can detect subtle voice abnormalities in subjects with HD. As impairment of phonatory function in HD was found to parallel increasing motor involvement, a qualitative description of voice dysfunction may be helpful to gain better insight into the pathophysiology of the vocal mechanism.

## Introduction

Huntington's disease (HD) is defined as an autosomal-dominant, progressive neuropsychiatric disorder caused by an expansion of the number of CAG repeats located on the short arm of chromosome 4 at 4p16.3 [1], [2]. From a clinical perspective, HD is mainly manifested by involuntary movements termed chorea, as well as psychiatric disturbances and cognitive deficits resulting in dementia [3]. Dystonia or rigidity may also manifest in some cases and stages of the disease. Moreover, patients with HD also develop a motor speech disorder characterized as hyperkinetic dysarthria in the course of illness, which occurs mainly as a consequence of underlying choreatic movements [4]. The most prominent signs of speech deviations in HD include phonatory dysfunction, unpredictable breakdowns of articulation, and abnormalities in speech timing and prosody [4], [5].

The quality of speech performance in HD is negatively affected by the involuntary contractions of vocal muscles, especially if there is a requirement for steady function. To this extent, the vocal task of sustained vowel phonation is particularly suitable because it demands stable coordination of the jaw, tongue, palate, and facial movements. Therefore, to assess changes of speech in the course of HD progression, the measurement of steady vowel prolongation is irreplaceable, providing the possibility to observe fluctuations induced by involuntary movements.

Previously, HD patients have been described by the presence of harsh, breathy, and strained-strangled voice with occasional pitch fluctuations and vocal arrests [6]–[9]. Furthermore, the severity of dysarthria seems to be related to the overall severity of motor symptoms in HD [7], [8]. Interestingly, preliminary reports have also suggested that speech deficits may precede the onset of the first motor symptoms [10], [11]. Considering the potential for early treatment and management strategies in HD due to its genetic predictability [12], objective clinical markers such as speech may be helpful in providing sensitive, quantitative information regarding treatment efficacy and disease onset/progression. Prior to investigating the suitability of acoustic analyses as an instrument for voice monitoring, the most prominent phonatory characteristics of HD patients should be well documented.

Thus, the aim of the current study was to examine mechanisms of HD-related phonatory dysfunction. Using the statistical classification model, another aim of the present investigation was to determine the optimal combination of objective phonatory parameters; establishing the most salient patterns of voice disorders in HD and the best separation of HD and healthy subjects. An additional aim was to find possible relationships between the phonatory parameters and duration and severity of HD.

## Methods

### Subjects

A total of 34 Czech native participants (15 men, 19 women), mean age 45.2± SD 13.3 (range 23–67) years, with genetically verified HD were recruited for the study. The mean age at HD onset was 39.3±13.5 (14–62) years, mean disease duration 5.9±3.1 (2–16) years, and average number of CAG triplet repeats 46.4±5.8 (40–70). Most of the patients (27/34) were treated in monotherapy or combination of benzodiazepines, antipsychotics, amantadine, and antidepressants. None of the HD participants had an occurrence of chronic obstructive pulmonary disease, asthma, allergy, infection of respiratory system, facial paresis of cranial nerves, or other symptoms unrelated to HD, which could negatively affect the patients' vocal performance. Each HD patient was assessed by a specialist in movement disorders using the Unified HD Rating Scale (UHDRS) [13].

As a healthy control (HC) group, we recruited 34 subjects (15 men, 19 women) of comparable age, mean age 45.5±13.6 (range 24–68) years, with no history of neurological or communication disorders. There were no significant differences in age distribution between the HD and HC groups. None of the HD or HC participants had undergone voice therapy. The study was approved by the Ethics Committee of the General University Hospital in Prague, Czech Republic and all participants provided written, informed consent to the vocal tasks and recording procedure.

### Speaking Tasks and Procedure

Speech data were recorded in a quiet room with a low ambient noise level using a head-mounted condenser microphone (Beyerdynamic Opus 55.09 Mk II SC, Heilbronn Germany) placed approximately 5 cm from the mouth. Voice signals were sampled at 48 kHz with 16-bit resolution. All subjects were recorded during a single session with a speech specialist. All participants were asked to perform sustained phonation of the vowel/a/and the vowel/i/, each repeated two times. These specific vowels were selected to be compatible with the most commonly used vowels in previous research [6], [9], [14]. All subjects were instructed to take a deep breath and produce vowel phonation at a comfortable pitch and loudness, as constant and long as possible. No time limits were imposed during recordings.

Quantitative acoustic analyses were designed using 10 traditional parameters including maximum phonation time (MPT), number of voice breaks (NVB), degree of voicelessness (DUV), fundamental frequency variations (F0 SD), recurrence period density entropy (RPDE), pitch period entropy (PPE), jitter, shimmer, harmonics-to-noise ratio (HNR), and detrended fluctuation analysis (DFA). In addition, two new parameters were introduced; the MPT until the first voice break (MPT_VB_) and the mel-frequency cepstral coefficient (MFCC). Together, these 12 parameters represent several specific aspects of phonatory dysfunction including airflow insufficiency, aperiodicity, irregular vibrations of vocal folds, signal perturbations, increased noise, and articulation deficiency [14]–[19]. A detailed description of each measurement can be found in Table 1. All acoustic parameters were designed to be gender-independent and to provide reliable automated assessment under practical conditions [14]. Gender distribution showed no significant differences between male and female participants across all variables. Test-retest reliability was assessed across the first and second cycle of each vowel and was found to be satisfactory (*r* = 0.83–0.93, *p*<0.001).

**Table 1:** Overview of the phonatory measurements applied to sustained vowel phonations.

Abbreviation		Description
**Airflow insufficiency**		
MPT (s)	Maximum phonation time	The aerodynamic efficiency of the vocal tract measured as the maximum duration of
		the prolonged vowel. This measure includes all voice breaks occurring during the entire
		vowel phonation. See Ramig et al. [6] for more information on MPT.
MPT_VB_ (s)	MPT until voice break	Maximum duration of the prolonged vowel until the first occurrence of voice break,
		present after at least 250 ms of modal phonation.
**Aperiodicity**		
NVB (−)	Number of voice breaks	Overall count of voice breaks. A voice break is defined as the distance between
		consecutive pulses longer than 1.25 divided by the bottom of the pitch range. The
		segment was defined as a voice break only if it occurred after at least 250 ms of modal
		phonation and 250 ms preceding the termination of phonation. Voice breaks can be
		associated with both low frequency drop and vocal arrest.
		See Boersma and Weenink [15] for further description.
DUV (%)	Degree of voicelessness	The fraction of pitch frames marked as unvoiced. A frame was considered unvoiced if
		it had voicing strength below the voicing threshold of 0.45 (autocorrelation function).
		See Boersma and Weenink [15] for further description.
**Irregular vibrations of vocal folds**		
F0 SD (st)	Standard deviation of	The variation in frequency of vocal fold vibration. The F0 sequence was converted to
	fundamental frequency (F0)	a semitone scale to avoid differences in gender. See Rusz et al. [14] for further description.
RPDE (−)	Recurrence period density	The ability of the vocal folds to sustain simple vibration. RPDE quantifies the deviations
	entropy	from periodicity, representing the uncertainty in the measurement of the pitch period.
		See Little et al. [16] for further description.
PPE (−)	Pitch period entropy	The inefficiency of voice frequency control. PPE uses the log-transformed linear
		prediction residuals of the pitch sequence in order to smooth vibrato. See Little et al. [17]
		for further description.
**Signal perturbations**		
Jitter (%)	Frequency perturbation	The extent of variation of the voice range. Jitter is defined as the variability of the
		fundamental frequency of speech from one cycle to the next.
		See Boersma and Weenink [15] for further description.
Shimmer (%)	Amplitude perturbation	The extent of variation of expiratory flow. Shimmer is defined as the
		sequence of maximum extent of the signal amplitude within each vocal cycle.
		See Boersma and Weenink [15] for further description.
**Increased noise**		
HNR (dB)	Harmonics-to-noise ratio	The amount of noise in the speech signal, mainly due to incomplete vocal fold
		closure. HNR is defined as the amplitude of noise relative to tonal components in
		speech. See Boersma and Weenink [15] for further description.
DFA (−)	Detrended fluctuation	The extent of turbulent noise in the speech signal. DFA measures the stochastic
	analysis	self-similarity of the noise caused by turbulent airflow through the vocal folds.
		See Little et al. [16] for further description.
**Articulation deficiency**		
MFCC (−)	Mel-frequency cepstral	Subtle changes in the motion of the articulators (jaw, tongue, lips). The MFCC
	coefficient	represents the vocal tract transfer function reflecting potential problems in the
		articulators. The MFCC parameter here was defined as the mean of the standard
		deviations of the 1st–12th MFCCs. It was designed to represent overall stability of
		individual vocal tract elements, as the individual MFCCs overlap the partitions of the
		frequency domain. The 1st–12th MFCCs were extracted using the implementation of
		Brooke's [18] Matlab toolbox with standard settings. See also Fraile et al. [19] for more
		information on MFCCs.

### Motor Symptoms

To assess the relationship between vocal and motor symptoms, voluntary (oculomotor and bradykinesia/fine motor) and involuntary (rigidity, dystonia, and chorea) components of the UHDRS motor score were assessed separately [20], [21]. In addition, the relationships between voice parameters and individual involuntary components of UHDRS were also investigated, where *rigidity* represents elevated muscle tone felt by the patient as muscle tension or spasm and by the examiner as increased resistance to passive movement across the joints; *chorea* is a state of excessive, spontaneous movements, irregularly timed, non-repetitive, randomly distributed and abrupt in character; and *dystonia* is involuntary sustained muscle contraction cause twisting and repetitive movements or abnormal postures.

### Statistics

Prior to statistical comparisons, average values for each acoustic parameter were calculated across all participants. Group differences were evaluated using analysis of variance (ANOVA) with post-hoc Bonferroni adjustment, as acoustic variables were normally distributed (Kolmogorov-Smirnov test). The Pearson correlation coefficient was applied to find relationships between variables. The level of significance was set at *p*<0.05.

To find the best combination of phonatory measurements for separating the HD and HC groups, we performed a classification experiment. First, a feature vector consisting of all 12 acoustic variables and all phonations (136 phonations for HD and 136 phonations for HC) was constructed. Subsequently, an exhaustive search for all possible combinations was performed using the method from statistical learning theory called support vector machine (SVM) [22]. On the basis of the decision boundary, the SVM classifier allows the construction of a predictive model that classifies a subject as either HD or HC. Since the data does not need to be linearly separable, the SVM classifier with Gaussian radial basis kernel was chosen because it enables a smooth, curved decision boundary. The choice of optimal SVM parameters was determined by a grid search over a range of values [22]; the optimal SVM parameters were found to be close to the default settings (C = 2, *σ = *2). To validate the reproducibility of the method, a cross-validation scheme was used. The original data (272 phonations) were randomly separated into two subsets: a training subset composed of 80% of the data (218 phonations), and a testing subset composed of 20% of the data (54 phonations). This process was repeated twenty times for each combination. The final performance of the model was calculated as the average percentage of correctly classified persons into proper groups (HD *vs*. HC) over all twenty cycles.

## Results

The mean UHDRS motor score was 29.5± SD 14.7 (range 3–70). According to the UHDRS speech item, 8 patients showed normal speech (score of 0), 21 patients had reduced intelligibility (score of 1, no need to repeat speech performance to be understood), and 3 patients manifested severe dysarthria (score of 2–4). UHDRS motor evaluation was not performed in two patients.

Measures of phonatory function showed significant differences between HD and HC groups, confirming the deterioration of voice in HD patients across all designed parameters (Table 2). In the HD group, the MPT_VB_ negatively correlated with voluntary components of UHDRS motor assessment score (*r = *−0.47, *p*<0.01), whereas there were no other correlations between acoustic parameters and voluntary components of motor performance. On the other hand, the involuntary components of UHDRS score show positive correlations to F0 SD (*r = *0.45, *p* = 0.01), RPDE (*r = *0.44, *p*<0.05), and MFCC (*r = *0.52, *p*<0.01). Considering individual involuntary components, the UHDRS dystonia subscore was positively correlated with DFA (*r* = 0.40, *p*<0.05) and RPDE (*r* = 0.38, *p*<0.05). In addition, the UHDRS chorea subscore was positively correlated to MFCC (*r* = 0.38, *p*<0.05) and showed a trend of weak positive correlation to F0 SD (*r* = 0.33, *p*<0.06). Similarly, the UHDRS rigidity subscore correlated with MFCC (*r* = 0.39, *p*<0.05). No correlations were seen between any acoustic parameters and disease duration.

**Table 2:** Results of voice analyses in HD and HC subjects.

Parameter	Group				Effect size	
	HD		HC		(Cohen's *d*)	
	Mean ± SD	Range	Mean ± SD	Range	HD vs. HC	
**Airflow insufficiency**						
MPT (s)	8.35±6.38	0.37–24.32	22.15±6.43	10.30–37.98	−2.16***	
MPT_VB_ (s)	5.48±4.87	0.37–22.63	21.46±6.79	10.30–37.98	−2.70***	
**Aperiodicity**						
NVB (−)	4.78±10.90	0–50.8	0.42±0.99	0–3.75	0.56*	
DUV (%)	6.28±7.52	0–31.42	0.22±0.76	0–4.36	1.14***	
**Irregular vibrations of vocal folds**						
F0 SD (st)	1.44±1.12	0.31–4.14	0.31±0.12	0.15–0.65	1.43***	
RPDE (−)	0.39±0.10	0.19–0.56	0.24±0.04	0.16–0.34	1.89***	
PPE (−)	0.40±0.20	0.11–1.08	0.21±0.12	0.06–0.57	1.10***	
**Signal perturbations**						
Jitter (%)	1.22±0.99	0.27–4.61	0.62±0.34	0.17–1.73	0.80**	
Shimmer (%)	6.36±3.70	2.26–17.61	3.99±1.80	1.75–9.61	0.81**	
**Increased noise**						
HNR (dB)	18.29±5.27	5.68–25.27	22.46±2.97	16.63–28.53	0.98***	
DFA (−)	0.63±0.02	0.60–0.70	0.62±0.01	0.59–0.64	0.97***	
**Articulation deficiency**						
MFCC (−)	0.59±0.13	0.35–0.90	0.38±0.04	0.31–0.48	2.16***	

* *p*<0.05;

** *p*<0.01;

*** *p*<0.001.

MPT = maximum phonation time, MPT_VB_ = maximum phonation time until first break, NVB = number of voice breaks, DUV = degree of voicelessness, F0 SD = variability of fundamental frequency, RPDE = recurrence period density entropy, PPE = pitch period entropy, HNR = harmonics-to-noise ratio, DFA = detrended fluctuation analysis, MFCC = mel-frequency cepstral coefficient.

**Figure 1:**
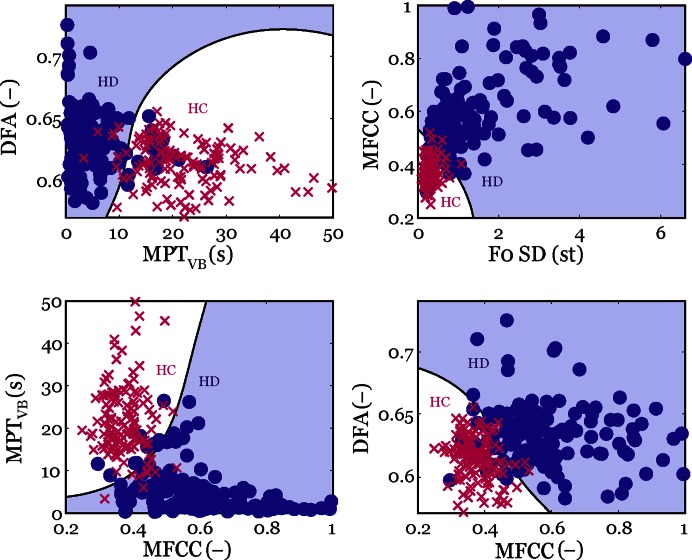
Selected pairs of the phonatory measures with classification boundary separating HD and HC subjects.

**Figure 2:**
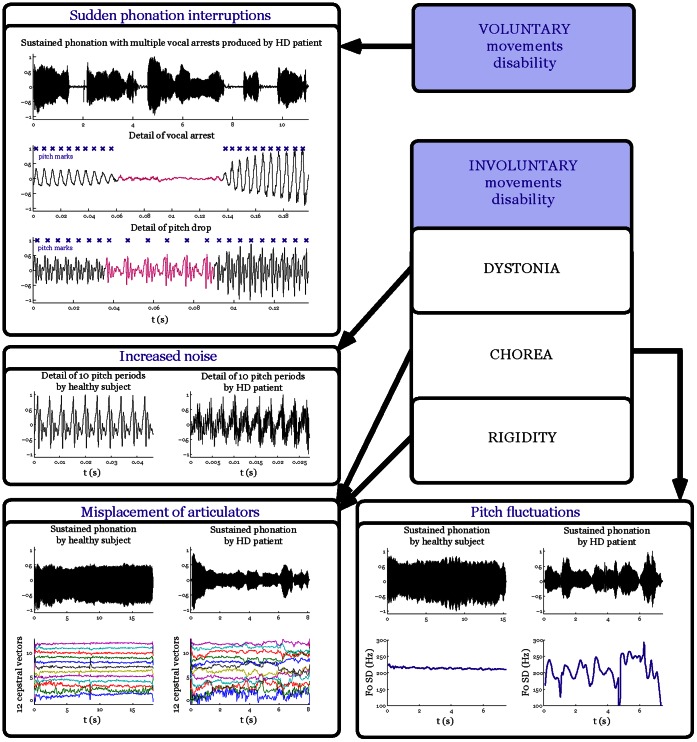
Scheme depicting the most salient features of dysphonia in HD and their relationship to motor symptoms.

Using the statistical model, we found that four aspects of voice measured by F0 SD (irregular pitch fluctuations), MPT_VB_ (sudden phonation interruption), DFA (increased noise), and MFCC (misplacement of articulators) can be considered as the most salient patterns of HD-related phonatory dysfunction. The combination of these 4 dysphonia measures leads to the best classification performance (94.1±2.3%) in discriminating HD patients from HC participants, with both exhibiting similar sensitivity (95.1±4.0%) and specificity (93.2±4.3%). Figure 1 shows the selected pairs of phonatory patterns with a complex boundary characterized by a specific curvature that allows the differentiation between HD and HC groups. The most frequent occurrence of single phonatory dysfunction was related to MPT_VB_ (89.4±3.7%; sensitivity 91.7±4.8%; specificity 87.8±5.2%) and F0 SD (84.9±4.3%; sensitivity 92.3±4.7%; specificity 80.2±5.6%); these phonation interruptions and pitch fluctuations were rarely observed in the performance of the healthy subjects. As can be seen from the results, the specificity is significantly decreased when considering only one aspect of phonatory dysfunction, whereas the overall performance for recognition of specific HD voice dysfunction is increased if several of the phonatory aspects are combined. Figure 2 summarizes the four main phonatory patterns in HD and their relationship to motor manifestations.

## Discussion

In the current study we investigated novel and traditional characteristics of phonatory dysfunction in HD using objective acoustic analyses. In contrast to previous studies that mostly focused on only one of the distinctive patterns of phonatory function, we examined several different aspects of voice in order to find most discriminative signs of dysphonia in HD. In the course of this study, we collected 136 phonations from 34 subjects with HD for comparison with 136 phonations of 34 control subjects; to the best of our knowledge, the largest series of data concerning phonatory deficits in HD. According to our findings, the main indicators of disrupted phonatory function in HD patients include sudden phonation interruptions, irregular pitch fluctuations, increased noise caused by turbulent airflow through the vocal folds, and subtle misplacement of articulators. We were able to predict HD group membership with 94% accuracy using these four dysphonia patterns and advanced statistical modeling. Furthermore, the best classification score was achieved using two newly designed measurements, MPT_VB_ and MFCC, resulting in a significant performance increase. Finally, we have shown that voice deficits in HD are related to both voluntary and involuntary motor disability.

Our findings are in accordance with previous studies reporting voice in HD patients as harsh with increased pitch breaks and fluctuations [6]–[9]. Generally, disturbances in HD vocalization are hypothesized to be a consequence of chorea, arising from dysfunction of the basal ganglia in HD. One common manifestation of voice dysfunction in HD is reduced maximum phonation time, due to airflow insufficiency [6], [7]. Shorter phonations can be accompanied by voice breaks associated with abrupt drops in fundamental frequency over a short period of time (lower frequency segments), as well as complete vocal arrests. Vocal breaks are hypothesized to be a consequence of abnormal muscle tone, hyper-adduction of the vocal folds, or stronger choreatic contractions of the laryngeal muscles leading to an abrupt end of phonation [6], [23], [24]. In fact, sudden phonation interruptions can be associated with motor impersistence, which is the inability to sustain certain simple voluntary acts such as maintaining a firm grip (milkmaid grip), or keeping the tongue protruded (darting tongue). However, there are somewhat contradictory reports in the literature regarding voice breaks and maximum phonation time [8], [9]. According to our findings, we were able to confirm reduction of phonation times and the presence of voice breaks. Moreover, we have noted an additional feature of HD vocal dysfunction that can be detected by the measurement of maximum phonation time until the first occurrence of voice break. This feature significantly contributed to the discrimination between the vocalizations of HD and healthy subjects, and therefore should be included in future HD-related speech studies. On the other hand, we could not agree with the conclusion that normal voices should not exhibit voice breaks during phonation [7], since we have occasionally observed short pitch drops in wider norm of healthy voices. Nevertheless, these pitch drops, if any, occur after a long period of phonation, whereas HD speakers frequently show pitch drops during even the first seconds of phonation.

The results of this study further indicate that HD voices manifest a considerably higher variability of fundamental frequency, which is consistent with previous research [9]. Pitch fluctuations are assumed to occur as a consequence of inefficient nervous system control, leading to sudden changes in laryngeal muscle tone. However, pitch fluctuations are evident only if the speaker is able to produce phonation for a longer period of time, which may not be possible in HD patients with severe dysarthria. Subsequently, in agreement with our results, increased noise in the speech signal is considered a common sign of phonatory dysfunction in HD [6]–[9], [24]. The noise components in speech can be caused by uncontrolled movements of laryngeal muscles and incomplete vocal fold closure, leading to inaccuracies in vibratory periods. Finally, subtle misplacement of articulators, a new and highly relevant clinical sign of voice dysfunction in HD seems to be associated with abnormal vocal tract configuration during phonation that can be satisfactorily captured by changes in the spectral domain. The individual spectral changes can be influenced by potential problems in the coordination of articulators, including misplacement of face, tongue, lips, and jaw.

One further aim of the current study was a comparison between phonatory variables and severity and duration of the disease. In agreement with previous studies [6]–[8], our results confirm that vocal dysfunction in HD seems to evolve with overall disease disability. The severity of HD dysphonia appears to be influenced by both voluntary and involuntary motor disability. According to our findings, it could be hypothesized that severe voluntary motor involvement is responsible for adductory phonatory terminations causing sudden stops or voice breaks. Further findings related to involuntary movements suggest that dystonia mainly contributes to harshness and strained-strangled voice quality, chorea to increased pitch fluctuations, and both chorea and rigidity are partially responsible for misplacement of articulators. Although involuntary movements, particularly chorea, predominate in the initial and middle stages of HD and may be replaced by rigidity in the later stages of HD [25], both chorea and rigidity can, in their own way, negatively affect the configuration of the vocal tract. In contrast, subtle impairment of voluntary movements such as sudden phonatory interruptions can already be presented in preclinical stages [26], where HD patients manifest deficits in planning, aiming, tracing and movement termination, as well as impaired initiation (akinesia) and slowness (bradykinesia) of movements [27], [28]. In summary, there is a large body of evidence that the severity of laryngeal dysfunction in HD correlates with the degree of motor impairment.

In general, abnormalities in voice and speech accompanied by other manifestations can raise suspicion about the etiology of the disease, and therefore the precise recognition of the type and severity of dysarthria may be essential for an accurate differential diagnosis [4], [5], [29], [30]. Comparing the current findings in hyperkinetic dysarthria to other fundamental types of dysarthria (hypokinetic, spastic, ataxic and flaccid), the only commonly documented phonatory feature in all dysarthrias is decreased quality of voice (breathiness, harshness, hoarseness, or strained-strangled voice) [4], [5], [31]. The higher incidence of voice breaks seems to occur only in spastic dysarthria [4], [5], whereas increased pitch variations were also reported in ataxic speakers [31], [32]. In addition, slight misplacement of articulators during phonation has also recently been shown in hypokinetic dysarthria [33], but we could not exclude the same behavior in other types of dysarthria as such a pattern has not been investigated.

Another potential application of voice and speech analyses in HD is related to early detection. Using a similar approach, we have shown previously that speech and voice disorders are detectable in at least 85% of parkinsonian patients at the time of their diagnosis [34]. More recently, Postuma et al. [35] investigated prodromal parkinsonism motor changes in idiopathic REM sleep behavior disorder and revealed voice and face akinesia as the earliest indicator of parkinsonism, with an estimated prodromal interval of 9.8 years before diagnosis. Accordingly in HD, there is also growing evidence that subtle neuropathological changes in cognitive, psychomotor, and behavioral aspects occur in HD individuals several years before diagnosis and manifestation of the first symptoms [12], [36]. Ramig et al. [24] were the first to investigate the stability of laryngeal musculature during phonation in twenty individuals at risk of developing HD. They found a twelve times greater incidence of pitch drops in subjects at risk of developing HD when compared to healthy speakers. Subsequently, Coleman et al. [37] revealed significant differences related to oral motor efficiency between individuals at risk of developing HD and healthy controls. Recently, several studies based on qualitative acoustic description have noted alterations in the vocal function of presymtomatic HD, mainly related to the timing of speech [10], [11]. However, the most salient phonatory aspects presented here were not included in their analysis. Despite the fact that approximately one quarter of the HD subjects in the present study exhibited normal speech performance according to the UHDRS speech item, we were able to discriminate huntingtonian from healthy speakers with 94% accuracy, supporting the hypothesis that subtle phonatory abnormalities are early and predominant manifestation of vocal impairment in HD.

One limitation of the current study is that the majority of our patients was treated by various drugs, and therefore the possible influence of medication on vocal performance cannot be excluded.

In conclusion, the present study illustrates the potential of voice analyses to document the degree and pattern of dysarthria in HD. Although speakers with HD manifested deterioration in all investigated voice measurements, the most prominent patterns of dysphonia were related to irregular pitch fluctuations, sudden phonation interruption, increased noise, and the misplacement of articulators. Since phonatory dysfunction in HD was found to parallel increasing motor involvement, a qualitative description of voice dysfunction may be helpful to gain better insight into the pathophysiology of the vocal mechanism.
