# Correction: Objective Acoustic Quantification of Phonatory Dysfunction in Huntington's Disease

**DOI:** 10.1371/annotation/a1ee87ea-1fdb-4baa-a8b0-62a44342f3cc

**Published:** 2013-09-13

**Authors:** Jan Rusz, Jiří Klempíř, Eva Baborová, Tereza Tykalová, Veronika Majerová, Roman Čmejla, Evžen Růžička, Jan Roth

In the Funding Statement, the number of the grant from the Czech Ministry of Health is incorrect. The correct number is: NT 12288-5/2011. 

